# Transthoracic Echocardiography in Assessing Patients with Suspected Infective Endocarditis (TEASE): An Exploratory Study

**DOI:** 10.3390/jcm14072195

**Published:** 2025-03-24

**Authors:** Nello Cambise, Saverio Tremamunno, Angelo Giuseppe Marino, Ludovica Lenci, Fabio De Benedetto, Antonietta Belmusto, Lorenzo Tinti, Antonio Di Renzo, Federico Di Perna, Giacomo Buonamassa, Sara Pontecorvo, Antonio De Vita, Massimiliano Camilli, Francesca Augusta Gabrielli, Francesca Graziani, Priscilla Lamendola, Gabriella Locorotondo, Rosaria Natali, Antonella Lombardo, Gaetano Antonio Lanza

**Affiliations:** 1 Dipartimento di Scienze Cardiovascolari e del Torace, Università Cattolica del Sacro Cuore, 00168 Rome, Italyangelomarino1995@gmail.com (A.G.M.); antonietta.belmusto@gmail.com (A.B.);; 2 Fondazione Policlinico Universitario A. Gemelli IRCSS, 00168 Rome, Italy; tremamunnosaverio@gmail.com (S.T.); francescaaugusta.gabrielli@policlinicogemelli.it (F.A.G.); gabryloc@hotmail.it (G.L.);

**Keywords:** infective endocarditis, transthoracic echocardiography, transesophageal echocardiography

## Abstract

**Background**: An extensive use of transesophageal echocardiography (TEE) has recently been suggested for the diagnosis of infective endocarditis (IE). In this study, we investigated whether among patients with negative transthoracic echocardiography (TTE), subgroups can be identified among whom TEE can be avoided/delayed. **Methods**: We conducted a retrospective study of 637 consecutive patients who underwent TEE for suspected IE. We selected 375 patients with negative TTE. For each patient, we obtained age, sex, blood culture (BC), blood exams, evidence of embolism, presence of moderate/severe heart valve disease, valve prostheses, and intracardiac devices. **Results**: IE was eventually diagnosed in 56 patients. Variables independently associated with IE at multivariate analysis included positive BC (OR 3.45; *p* = 0.006), evidence of embolism (OR 13.0; *p* < 0.001), bioprosthetic heart valves (OR 4.31; *p* < 0.001) and platelet count < 150,000/mL (OR 2.47; *p* = 0.014). In patients without any of these predictors for IE (*n* = 81), only 1 had a diagnosis of IE and no in-hospital IE-related deaths occurred. Among patients with negative BC (*n* = 127), IE prevalence increased with the number of other predictors, but IE-related mortality was 0%. IE prevalence (10.8%) and IE related in-hospital mortality (2.7%) were also rather low in patients with a positive blood culture without any other independent predictors for IE but were 20% (IE-related mortality 3.8%) and 71% (IE-related mortality 28.6%) in those with only one or 2–3 other IE predictors, respectively. **Conclusions**: Our data suggest that, among patients with suspected IE and negative TTE, subgroups can be identified in whom TEE might be safely avoided or delayed.

## 1. Introduction

Infective endocarditis (IE) represents a significant public health concern as it is associated with elevated morbidity and mortality rates, posing substantial challenges for healthcare systems in terms of prevention, diagnosis, and management [1,2]. The incidence of IE has been estimated to be around 14/100,000 people a year, which has apparently been increasing over the last decade, and was responsible for around 66,300 deaths globally in 2019 [3].

The most recent ESC guidelines suggest that IE should be suspected in all patients with sepsis or a fever of unknown origin, especially in patients with one or more risk factors, but in some patients it might present with less specific clinical symptoms [3].

Echocardiography plays a central role in the diagnosis of IE, with transesophageal echocardiography (TEE) showing a higher diagnostic accuracy compared to transthoracic echocardiography (TTE) [4,5]. Recent guidelines suggest that TEE should be performed in all patients with suspected IE, with the only exception being in unequivocal cases of isolated IE of the tricuspid valve [3]. Specifically, guidelines recommend TEE “in all patients with clinical suspicion of IE and a negative or non-diagnostic TTE” (Class IB) [3].

Previous studies, in fact, showed that TEE has a higher diagnostic accuracy for IE than TTE. However, the comparisons between the two methods were usually based on echocardiographic findings only (mainly, presence and characteristics of suspected valve vegetations) [6,7,8,9]. There are limited data, instead, on the specific assessment of the negative predictive role of TTE for IE with echocardiographic findings integrated with clinical findings of patients [10,11,12].

Thus, the purpose of this study was to investigate whether, among patients with suspected IE who show a negative TTE, subgroups at low risk, in which the diagnosis of IE can be reasonably excluded and TEE avoided or safely delayed, as well as subgroups at high risk, in which TEE should instead be performed as soon as possible, can be identified.

## 2. Methods

### 2.1. Patients

We retrospectively identified all patients who underwent TEE for suspected IE at the echocardiography laboratory of the University Hospital Agostino Gemelli in Rome, Italy, between January 2021 and December 2023. Patients were included in this study if they fulfilled the following inclusion criteria: (1) they had undergone TTE at our echo-lab within 14 days prior to TEE; and (2) they had a negative TTE, i.e., they showed no evidence of findings indicative of, or suspected for, IE.

IE is a heterogeneous disease with highly variable clinical presentations; therefore, the suspicion of IE was always at the discretion of the attending physicians, based on various clinical settings, including sepsis conditions, persistent fever, high white blood count and inflammatory indexes, or unexplained embolic events.

Patients were excluded if they had a positive/doubtful TTE, had a history of previous IE, were transferred with a definite diagnosis of IE from another hospital, were not hospitalized, or had TTE performed more than 14 days before TEE. Patients with poor echocardiographic windows that did not allow a reliable assessment of cardiac abnormalities (inconclusive TTE) were also excluded. Image quality was based on the ability to clearly visualize all cardiac valves, chambers, and prosthetic materials, allowing definitive identification or exclusion of vegetations, abscesses, or prosthetic valve dehiscence. Studies with inadequate visualization of these structures were classified as inconclusive and excluded from the analysis.

The following echocardiographic variables at the time of TTE were collected for each patient: presence of biologic or mechanic valve prosthesis or intracardiac device; evidence of moderate/severe valve disease (stenosis and/or insufficiency); and left ventricle ejection fraction (LVEF).

In addition, the following clinical variables were collected from our hospital electronic database: age, sex, body mass index (BMI), routine blood and chemistry tests, blood culture (BC) results, clinical evidence of concomitant infection, and evidence of systemic/pulmonary embolism. The study was approved by the Ethics Committee of our institution.

### 2.2. Endocarditis Diagnosis

The diagnosis of IE was made using the modified Duke’s criteria, that are described in detail elsewhere [3]. Accordingly, a “definite IE” was diagnosed by the presence of: (1) 2 major criteria (microbiologic and imaging); or (2) 1 major criterion and ≥3 minor criteria (predisposing conditions, fever with skin temperature >38 °C, embolism, immunologic phenomena, minor microbiologic evidence); or (3) all 5 minor criteria. A “possible IE” was instead diagnosed in the presence of: (1) 1 major criterion and 1–2 minor criteria; or (2) 3–4 minor criteria.

In patients with doubtful findings at the time of TEE, further evaluation (including repeating the TEE after a few days and/or performing 18F(fluoro)-deoxy-glucose positron emission tomography/computed tomography (FDG-PET/CT) depending on clinical judgment) was performed to obtain a final clinical diagnosis. Patients in whom a clear diagnosis of IE could not be achieved despite complete investigation, but IE remained a probable/possible diagnosis, were included among those with a final diagnosis of IE.

The diagnosis of IE during TTE and TEE was made due to the presence of vegetations, abscesses, and/or a new dehiscence of a prosthetic valve [3,13]. Vegetations with typical features (i.e., oscillating masses attached to a valvular structure, with a motion independent of that of the valve apparatus) were considered diagnostic for IE. Non-oscillating masses with an atypical location were considered doubtful for the diagnosis. TTE was considered negative in the absence of evidence of vegetations, abscesses, and prosthetic valve dehiscence.

### 2.3. Clinical Outcomes

In-hospital mortality related to IE was the primary outcome of the study. Death was considered to be related to IE in case of: (1) death from septic shock in the presence of a clear diagnosis of IE; (2) death from cardiogenic shock resulting from severe acute valvular disease caused by IE; (3) death occurring during or after heart surgery. In-hospital non-IE-related mortality (e.g., deaths resulting from non-cardiac infections or unrelated medical conditions) was also evaluated.

### 2.4. Statistical Analysis

Continuous variables are expressed as means with standard deviations. Discrete variables are reported as numbers and percentages. Comparisons between groups of nominal variables were performed using a Chi-square test. The risk of individual variables associated with IE was assessed by univariate logistic regression. Multivariable logistic regression analysis was applied to identify variables independently associated with IE. Only variables with *p*-values < 0.1 at standard statistical analysis were included in the multivariable analysis. A *p* value < 0.05 was always required for statistical significance. Statistical analyses were performed with the SPSS 28.0 software (SPSS Italia, Florence, Italy).

## 3. Results

### 3.1. Population

**Figure 1:**
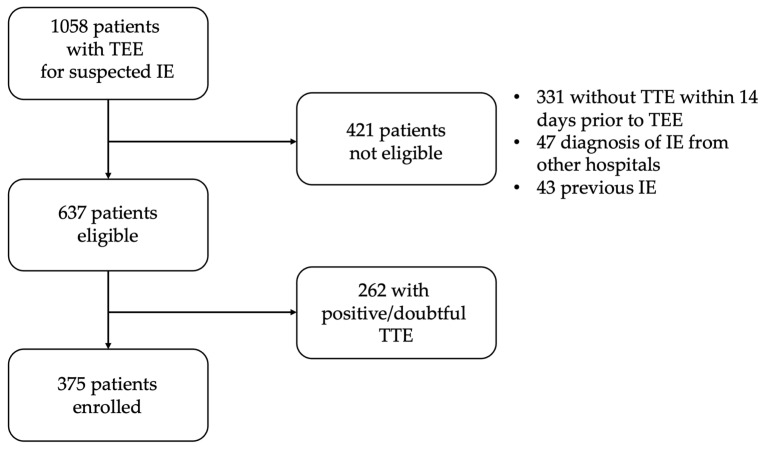
Flow-chart of patient selection. TTE: transthoracic echocardiogram; IE: infective endocarditis; TEE: transesophageal echocardiography.

During the study period, 1058 patients underwent TEE for suspected IE at our echocardiography laboratory. After excluding patients who did not have TTE performed within 14 days prior to their TEE, had a previous diagnosis of IE, or already had a diagnosis of IE performed in another hospital, 637 patients (57%) were considered eligible for the study. Of these patients, 262 (41%) had a positive or doubtful/inconclusive TTE for IE and were, therefore, excluded. Thus, 375 patients (59%) who had an unequivocal negative TTE for IE formed the population of this study (Figure 1). The median (interquartile range) time between TTE and TEE in these patients was 5.8 days (5.4–6.2).

#### 3.1.1. IE Diagnosis

Among the 375 patients with a negative TTE, the TEE was positive for IE in 44 (12%) and doubtful in 29 (8%) patients. After a second TEE and/or further diagnostic investigation, a final diagnosis of definite IE was reached in 49 patients (13.1%). Furthermore, a probable/possible diagnosis of IE still remained even after full investigation in another seven patients (1.9%), and these were included in the IE group. Therefore, 56 patients (15%) eventually formed the group of patients with IE in this study.

Table 1 shows the main clinical characteristics of patients with and without a final diagnosis of IE, whereas Table 2 summarizes the main echocardiographic findings of the two groups. Variables associated with IE included the presence of biological valve prostheses (*p* < 0.001), systemic/pulmonary embolism (*p* < 0.001), lower platelet count (*p* < 0.001), positive blood cultures (*p* = 0.003), moderate/severe aortic valve regurgitation (*p* = 0.022), moderate/severe mitral valve regurgitation (*p* = 0.022), high white blood cell count (*p* = 0.042), and low hemoglobin level (*p* = 0.048). Following its high relationship with IE, platelet count was also dichotomized based on the lowest normal level for our laboratory (150,000/mL). Platelet count < 150,000/mL showed also a high significant association with IE (*p* < 0.001; Table 1) and was used in subsequent analyses.

During multivariable logistic regression, independent predictors for IE included evidence of systemic or pulmonary emboli (OR [odds ratio] 13.2; *p* < 0.001), positive blood cultures (OR 3.79; *p* = 0.017), presence of bioprosthetic heart valves (OR 3.79; *p* < 0.001), and platelet count < 150,000/mL (OR 2.50; *p* = 0.014) (Table 3; central illustration).

#### 3.1.2. Subgroup Analysis

To obtain detailed data about the negative predictive role for IE of a negative TTE, we considered subgroups of patients according to various combinations of the presence or absence of the four independent predictors for IE.

Specifically, patients were divided into two groups according to the presence of negative or positive blood cultures, and in each group we defined subgroups according to the presence of zero, one, or two to three of the other three independent risk factors for IE. The main results concerning these subgroups of patients are summarized in Table 4 (central illustration). As shown, the negative predictive value (NPV) for IE was very high (>90%) among patients with negative blood cultures, except in those with two to three other independent risk factors. In particular, in patients without any independent risk factor for IE (*n* = 81; 21.6% of the population), the NPV for IE of a negative TTE was 98.8%. The NPV also seemed high (88.2%) in patients with positive blood cultures but no other risk factors for IE.

Among patients with a positive blood culture who were negative for IE (*n* = 201), the source of bacteremia included urinary tract infections in 30.3% of patients, respiratory tract infections in 17.4%, gastro-intestinal tract infections in 10.3%, and skin/soft tissue infections in 37.3%, whereas in 5.0% of patients the source of bacteremia was not clear.

#### 3.1.3. Clinical Outcomes

In-hospital IE and non-IE related mortality, together with heart surgery rate in the subgroups in which the population was divided, are also shown in Table 4. IE related mortality in the whole population of patients with negative TTE occurred in 13 patients (3.5%).

In-hospital global mortality was 14.1% (*n* = 53). Non-IE related mortality was higher than IE-related mortality in the whole population (10.7% vs. 3.5%, respectively). Furthermore, mortality was higher in patients with a positive blood culture vs. those with a negative blood culture (19% vs. 4.6%, respectively).

Notably, no IE related deaths and a very low rate of heart surgery were observed in the subgroups of patients with negative blood cultures, independently of the presence of other independent risk factors for IE. All deaths in this subgroup of patients, therefore, occurred for non-IE related causes and, accordingly, were not influenced by the presence of risk factors for IE (Table 4). Non-IE related mortality was also prevalent in patients with positive blood cultures (*n*= 34, 13.8%; 72.3% of all-cause mortality), with exception of the small subgroup of patients with two to three risk factors, in whom mortality was exclusively related to IE, in accordance with the importance of these factors.

Among patients with positive blood cultures, both IE-related death (2.7%) and heart surgery (3.4%) seemed to be low among the rather large group of patients without any other risk factor for IE (*n* = 148; 34% of the population).

## 4. Discussion

To the best of our knowledge, this is the first study to specifically assess how accurate a negative TTE can be in excluding infective endocarditis (IE) when interpreted within the context of a full clinical evaluation. Even more, our study correlates these diagnostic findings with in-hospital clinical outcomes. Overall, our data indicate that among patients with a clinical suspicion of IE who initially present with a negative TTE, it is possible to identify subgroups at low risk for both IE and IE-related death, in whom TEE may be safely avoided or delayed, and subgroups at higher risk, in whom more urgent investigation is warranted.

### 4.1. TTE Performance and Clinical Implication

Current guidelines recommend performing TEE in patients with a negative TTE when there is a clinical suspicion of IE [3]. Nevertheless, the concept of “clinical suspicion of IE” is largely individual and not standardized at present, which may sometimes result in a referral to TEE for patients with a very low risk profile for IE.

However, TEE is an uncomfortable exam, which may require deep sedation, and carries a small but non-negligible risk of complications such as laryngospasm, dysphagia, and—rarely—esophageal lesions or bleeding [14,15]. Avoiding TEE when unnecessary would, therefore, be highly desirable. Our data suggest that, among patients referred to echocardiographic laboratories for IE suspicion, a subset can in fact be identified, based on a negative TTE plus certain clinical and laboratory findings, in whom the likelihood of IE is extremely low. These patients may not require an immediate TEE, though close observation is needed to promptly detect any change in clinical conditions. If confirmed by larger prospective studies, our findings may encourage refinement of the current recommendations for TEE by highlighting specific low-risk scenarios in which it could be safely deferred.

### 4.2. Previous Evidence on Negative TTE

Several prior investigations have evaluated the negative predictive value (NPV) of TTE in excluding IE, generally confirming a valuable accuracy [7,16,17]. A meta-analysis of 16 studies involving 2807 patients showed an NPV ranging from 62% to 85%, varying with patient selection, criteria for TTE negativity, and echocardiographic imaging modalities [18]. In populations resembling ours—namely, those including patients with prosthetic valves, using harmonic imaging, and strictly excluding inconclusive or doubtful TTEs—this NPV approached 85%, closely mirroring our results. A subsequent meta-analysis of 2209 patients reported an average NPV of 78% (i.e., a 22% false-negative rate), which many clinicians would deem relatively high for a condition carrying the potentially severe consequences of IE [19]. Such data have historically supported performing TEE in virtually all patients with a negative TTE and clinical suspicion of IE.

Importantly, most prior studies focused on imaging criteria alone (i.e., the absence of TTE findings such as vegetations) without thoroughly integrating additional clinical risk markers. Lindner et al. [10] did suggest that a negative TTE could be reliable in excluding IE among patients with a low clinical probability; however, their study included only 67 and 14 patients with low- or intermediate-probability IE, respectively, and there was limited subgroup analysis aimed at identifying very-low-risk patients among those with a negative TTE. Our study expands on these observations by combining echocardiographic, clinical, and laboratory variables in a larger cohort to refine risk stratification more precisely.

### 4.3. Risk Stratification and Main Predictors of IE

Among patients with a strictly negative TTE, we identified four independent predictors of IE: (1) positive blood cultures; (2) presence of a bioprosthetic valve; (3) evidence of systemic/pulmonary embolism; and (4) a low platelet count (<150,000/μL). While the higher risk of IE with positive blood cultures is intuitive [20,21], the presence of bioprosthetic valves, whether implanted surgically or via percutaneous approach (i.e., TAVI), can pose significant diagnostic challenges for TTE due to acoustic artifacts and calcifications [22,23,24,25]. In addition, systemic or pulmonary emboli of unclear origin should heighten clinical suspicion of minor cardiac vegetations that may have eluded TTE [26,27,28]. Finally, thrombocytopenia aligns with previous studies showing that low platelet counts are predictive of both IE and worse outcomes [29,30,31].

In our cohort, mechanical valves showed a lower incidence of IE (9%) compared to bioprosthetic valves (37%). The lower frequency of IE in patients with mechanical valves observed in our study aligns with previous evidence suggesting that mechanical valves, despite being prone to thromboembolic events, may carry a lower risk of IE compared to bioprosthetic valves due to differences in the material surface properties and lower susceptibility to microbial colonization [25]. However, our data on mechanical valves must be taken with a lot of caution due to the very low number of patients with this type of prosthesis (*n* = 5) included.

Our subgroup analyses revealed a strikingly low incidence of IE—and no IE-related deaths—among patients with negative blood cultures, even if they had other risk factors. Conversely, patients with positive blood cultures but no additional risk factors still had a relatively small, though not negligible, risk of IE (10.8%), which increased considerably when one or more of the additional risk predictors were present (20.3% and 71.4%, respectively). These findings underscore how combining microbiological, clinical, and echocardiographic data can refine IE risk estimation far more accurately than TTE findings alone.

### 4.4. Clinical Outcomes and Implications for Clinical Practice

In-hospital IE-related mortality in our overall negative-TTE population was 3.5%.

Non-IE related mortality was prevalent compared with IE-related mortality, except for the subgroup with positive blood cultures and two to three risk factors. Notably, no IE-related deaths and very few IE-related surgeries were observed in patients with negative blood cultures, regardless of other risk factors. Even in patients with positive blood cultures, the risk of mortality and surgical intervention was still modest when no other risk factor was present, but it increased substantially with each additional risk factor. Confirming the importance of the identified risk factors was the consistent increase in the proportion of IE-related deaths with the increase in their number [29,30,31,32,33,34]. Among patients with positive blood cultures, indeed, IE-related deaths were only 17% and 18% of all deaths, whereas 100% of deaths were related to IE in patients with two to three risk factors (see Table 4).

Taken together, these data suggest that clinicians could adopt a more individualized approach to TEE indications. Patients exhibiting a negative TTE and none of these risk factors (especially with persistently negative blood cultures, which included 21.6% of our population), have a low probability of really having IE (only 1.2% has a final diagnosis of IE) and in-hospital mortality can be 0%; therefore, these patients may undergo meticulous clinical observation and repeat imaging if their status changes. Conversely, those harboring multiple risk factors or presenting with persistent bacteremia should undergo an immediate TEE to confirm or exclude IE. It has indeed been shown that delaying IE diagnosis is associated with a higher mortality rate [35,36] and risk of complications [37], suggesting that TEE might only be deferred in a very selected group of patients with a very-low probability of IE and very-low risk of IE related complications.

**Figure 2:**
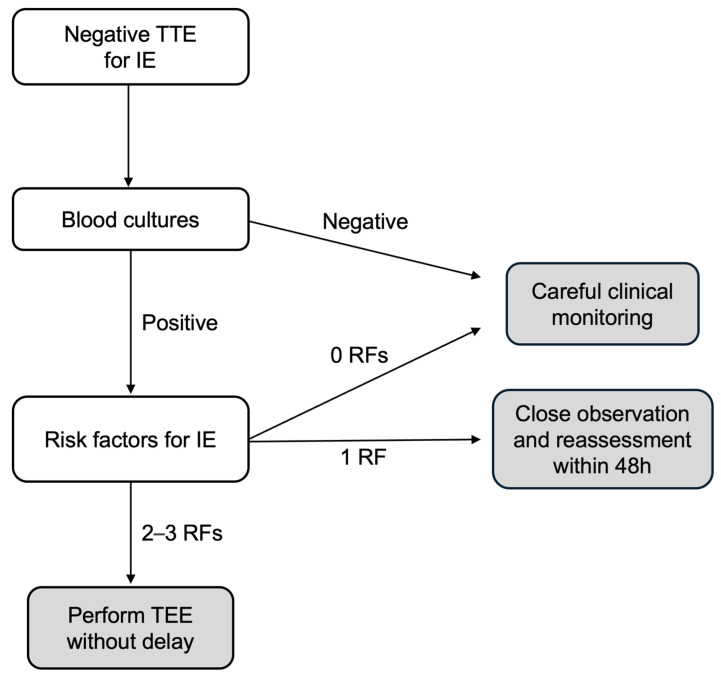
Flow-chart for TEE need/timing. TTE: transthoracic echocardiogram; IE: infective endocarditis; TEE: transesophageal echocardiography; RF: risk factor.

Particularly, based on the data emerging from our study, among patients with suspected IE but negative TTE, three subgroups may be identified that present a low, moderate, and high risk for IE (Table 4) and for which a tailored approach might be applied in clinical practice, as outlined in Figure 2. The groups are as follows:

(1) The low-risk group includes patients with negative BCs or positive BCs but no risk factors for IE; in these patients, TEE seems can be safely omitted but maintaining careful clinical monitoring;
(2) The moderate-risk group includes patients with positive BCs but only one risk factor for IE; in these patients, close observation and short-term reassessment (within 48 h) are recommended, and TEE should be considered based on clinical/echocardiographic changes;
(3) The high-risk group includes patients with positive BCs and two to three risk factors for IE; in these patients, TEE should be performed immediately.

It should be underscored, however, that our study included only a small number of prosthetic-valve cases (particularly, mechanical valves) and, therefore, our findings should be interpreted very cautiously in this subset, in which TEE should generally remain a high priority if any suspicion for IE persists despite a negative TTE. Accordingly, further studies are needed to confirm our results by including a larger proportion of this specific population.

## 5. Limitations of the Study

Some limitations of our study should be noted. Firstly, our study was retrospective, and therefore subjected to some inherent biases (e.g., selection, confounding, information, and observer biases), which may limit generalizability of results; thus, our findings need confirmation in larger prospective studies.

Secondly, the definition of suspected IE was not standardized; the indication for TEE was indeed at the total discretion of the attending physicians, who sometimes might have inappropriately referred patients for TEE; however, at present there are no standardized criteria on which clinicians can base their suspicion for IE and, therefore, our study reflects real-world practices.

Thirdly, the influence of some specific comorbidities, such as chronic respiratory failure or previous cerebrovascular events on IE risk or outcomes was not addressed and warrants future study.

Fourthly, as already noticed above, the number of patients with prosthetic valves (particularly, mechanical valves) was small and, therefore, further studies are particularly necessary in this subgroup of patients to validate data emerging from the present study.

Finally, we focused on initial TTE results and did not account for changes in clinical status that might have emerged later during hospitalization; thus, prospective studies should incorporate serial imaging and evolving clinical parameters in the assessment of the NPV for IE of a negative TTE. The latter point is relevant when considering that some risk for IE still persisted also in the low-risk subgroups of our patients, thus suggesting that close monitoring of clinical conditions should always be performed and TEE or more sophisticated diagnostic methods (i.e., fluorodeoxyglucose positron emission tomography/computed tomography) utilized as soon as the suspicion persists, even in these patients.

## 6. Conclusions

In conclusion, our findings show that a negative TTE, when integrated with key clinical and laboratory indicators, can achieve a high NPV for IE in selected subgroups of patients with suspected IE. If confirmed in larger prospective studies, this approach might have potential practical implications, as it might suggest that, in such very-low-risk patients, an immediate TEE may be safely avoided or deferred, sparing unnecessary procedures. Conversely, our data indicate that patients with multiple risk predictors or positive blood cultures deserve a prompt TEE to avoid missing or delaying the diagnosis of a potentially life-threatening disease.

## Figures and Tables

**Table 1:** Main clinical characteristics of patients with or without a final diagnosis of infective endocarditis.

	Infective Endocarditis (*n* = 56)	No Infective Endocarditis (*n* = 319)	Odds Ratio (95% CI)	*p*
Age	69 ± 13	67 ± 15	1.01 (0.99–1.03)	0.39
Male	37 (66%)	209 (66%)	1.03 (0.56–1.89)	0.94
Body mass index (Kg/m^2^)	56 ± 26	316 ± 25	1.01 (0.98–1.03)	0.57
Diabetes	20 (37%)	81 (25%)	1.63 (0.89–2.98)	0.11
Positive blood cultures	47 (84%)	201 (63%)	3.07 (1.45–6.48)	0.003
Gram-positive	44 (79%)	167 (80%)	3.43 (1.55–7.56)	0.002
Gram-negative	3 (5%)	34 (16%)	0.41 (0.12–1.40)	0.16
*Staph. Aureus*	16 (31%)	105 (39%)	0.70 (0.37–1.33)	0.28
*Staph. Epidermidis*	4 (8%)	14 (5%)	1.53 (0.48–4.85)	0.47
*Str. Viridans*	1 (2%)	2 (1%)	2.64 (0.24–29.63)	0.43
*Str. Gallolyticus*	4 (8%)	9 (3%)	2.43 (0.72–8.20)	0.15
*E. Faecalis*	9 (18%)	18 (7%)	2.93 (1.24–6.95)	0.015
HACEK	0 (0%)	1 (0.4%)	1.88 (0.08–46.71)	0.99
Fungal	1 (2%)	12 (4%)	0.42 (0.05–3.33)	0.41
Fever	37 (66%)	191 (60%)	1.30 (0.71–2.35)	0.40
Urinary tract infection	5 (9%)	61 (19%)	0.42 (0.16–1.08)	0.072
Respiratory tract infection	14 (25%)	47 (15%)	1.93 (0.98–3.81)	0.06
Gastro-intestinal tract infection	3 (5%)	31 (10%)	0.53 (0.16–1.78)	0.30
Skin/soft tissue infection	12 (21%)	102 (32%)	0.58 (0.29–1.15)	0.12
Embolism	21 (38%)	16 (5%)	11.36 (5.43–23.78)	<0.001
Hemoglobin (g/dL)	10.7 ± 1.7	11.5 ± 4.4	0.87 (0.75–0.99)	0.048
White blood cells (×10^9^/L)	13.8 ± 7.7	12.5 ± 6.4	1.83 (1.02–3.28)	0.042
Platelets (×10^9^/L)	191 ± 114	256 ± 137	0.99 (0.992–0.998)	<0.001
Platelets < 150 × 10^9^/L	21 (38%)	56 (18%)	2.82 (1.53–5.20)	<0.001
Creatinine (mg/dL)	1.28 ± 1.1	1.54 ± 1.7	0.88 (0.70–1.11)	0.28
Bilirubin (mg/dL)	0.94 ± 0.8	1.03 ± 1.56	0.95 (0.74–1.22)	0.68
C-reactive protein (mg/L)	142 ± 88	157 ± 202	0.99 (0.99–1.00)	0.58
Procalcitonin (ng/mL)	5 ± 10	10 ± 34	0.99 (0.97–1.01)	0.31

CI = confidence interval; *Staph.* = Staphylococcus; *Str.* = Streptococcus; *E.* = Enterococcus; HACEK = *Haemophilus*, *Aggregatibacter actinomycetemcomitans*, *Cardiobacterium Hominis*, *Eikenella Corrodens*, *Kingella Kingae*.

**Table 2:** Main echocardiographic characteristics of patients with or without a final diagnosis of infective endocarditis.

	Infective Endocarditis (*n* = 56)	No Infective Endocarditis (*n* = 319)	Odds Ratio (95% CI)	*p*
Days between TTE and TEE	5 ± 3	6 ± 4		0.075
LVEF (%)	54 ± 12	54 ± 11	1.00 (0.98–1.03)	0.94
Aortic regurgitation	11 (20%)	29 (9%)	2.44 (1.14–5.22)	0.022
Aortic stenosis	6 (11%)	17 (5%)	2.13 (0.80–5.65)	0.13
Mitral regurgitation	17 (30%)	54 (17%)	2.13 (1.12–4.04)	0.021
Mitral stenosis	2 (4%)	3 (1%)	3.89 (0.64–23.82)	0.14
Tricuspid regurgitation	14 (25%)	54 (17%)	1.63 (0.83–3.19)	0.15
Central venous catheter	7 (13%)	56 (18%)	0.67 (0.29–1.56)	0.35
Cardiac electronic device	15 (27%)	69 (22%)	1.33 (0.69–2.54)	0.39
Mechanical heart valve	5 (9%)	20 (6%)	1.47 (0.53–4.08)	0.46
Bioprosthetic heart valve	21 (37%)	44 (14%)	3.75 (2.00–7.03)	<0.001

CI = confidence interval; LVEF = left ventricle ejection fraction.

**Table 3:** Multivariable logistic regression analysis for prediction of infective endocarditis.

	Odds Ratio (95% Confidence Interval)	*p*
Systemic/pulmonary embolism	13.25 (5.49–31.98)	<0.001
Bioprosthetic valve	3.79 (1.76–8.16)	<0.001
Platelets < 150 × 10^9^/L	2.50 (1.20–5.22)	0.014
Positive blood cultures	3.79 (1.27–11.34)	0.017
Urinary tract infection	0.39 (0.13–1.12)	0.08
Respiratory tract infection	1.76 (0.76–4.07)	0.18
Hemoglobin (g/dL)	0.89 (0.75–1.07)	0.24
White blood cells (×10^9^/L)	0.92 (0.38–2.21)	0.85
Aortic regurgitation	1.74 (0.66–4.54)	0.26
Mitral regurgitation	2.01 (0.89–4.55)	0.09

**Table 4:** Risk of infective endocarditis and main outcomes according to negative or positive blood cultures and presence of other independent risk factors for endocarditis.

	No. Patients	% of Patients	IE Diagnosis	Risk for IE			IE-Related Mortality	Non IE-Related Mortality	Heart Surgery
				OR	CI 95%	*p*			
Negative BCs	127	34%	9 (7.1%)	1.00 *	-	-	0 (0%)	6 (4.6%)	2 (1.6%)
0 RF	81	22%	1 (1.2%)	1.00 ^†^	-	-	0 (0%)	3 (3.7%)	1 (1.2%)
1 RF	38	10%	3 (7.9%)	1.2	0.28–5.00	0.82	0 (0%)	3 (7.9%)	1 (1.2%)
2–3 RF	8	2%	5 (62.5%)	48.0	8.37–275	<0.001	0 (0%)	0 (0%)	0 (0%)
Positive BCs	248	66%	47 (19%)	3.1	1.45–6.48	0.003	13 (5.2%)	34 (13.8%)	13 (5.2%)
0 RF	148	39%	16 (10.8%)	1.6	0.68–3.73	0.29	4 (2.7%)	20 (13.5%)	5 (3.4%)
1 RF	79	21%	16 (20.3%)	3.3	1.39–7.96	0.007	3 (3.8%)	14 (17.7%)	5 (6.3%)
2–3 RF	21	6%	15 (71.4%)	33.0	10.2–105	<0.001	6 (28.6%)	0 (0%)	3 (14.3%)

BCs = blood cultures; CI = confidence interval; IE = infective endocarditis; OR = odds ratio; RF = independent risk factors for infective endocarditis on multivariable logistic regression, including heart valve bioprostheses, embolism, platelet count < 150,000/mL. * Reference for the risk of positive BCs vs. negative BCs; ^†^ reference for the risk of subgroups with 0, 1 and 2–3 risk factors for IE.

## Data Availability

The data presented in this study are available upon request from the corresponding author.

## Abbreviations

The following abbreviations are used in this manuscript:

IE	Infective Endocarditis
TTE	Transthoracic Echocardiography
TEE	Transesophageal Echocardiography
BC	Blood Culture
LVEF	Left Ventricular Ejection Fraction
